# P-725. Incidence and Severity of Influenza-like Illness in Cigarette Users: A Prospective Study

**DOI:** 10.1093/ofid/ofae631.921

**Published:** 2025-01-29

**Authors:** Rachael D C Jones, Kat Schmidt, Christina Schofield, Anuradha Ganesan, Wesley Campbell, David Hrncir, Tahaniyat Lalani, Tyler Warkentien, Katrin Mende, Ana E Markelz, Catherine Berjohn, Laurie Housel, Jitendrakumar Modi, Adam Saperstein, Alan Williams, Bruce McClenathan, Limone Collins, Christina Spooner, Srihari Seshadri, Ryan C Maves, Gregory Utz, Robert O’Connell, Mark Simons, Simon Pollett, Christian L Coles, Rhonda E Colombo, Timothy Burgess, Stephanie A Richard

**Affiliations:** USUHS, Bethesda, Maryland; HJF, Bethesda, Maryland; Madigan Army Medical Center, Tacoma, Washington; Infectious Disease Clinical Research Program, USUHS; Henry M. Jackson Foundation for the Advancement of Military Medicine Inc, Bethesda, Maryland; Walter Reed National Military Medical Center, Bethesda, Maryland; Carl R. Darnall Army Medical Center, Fort Cavazos, Texas; Naval Medical Center Portsmouth, Portsmouth, Virginia; Naval Medical Center Portsmouth, Portsmouth, Virginia; Infectious Disease Clincial Research Program, JBSA Ft Sam Houston, Texas; Brooke Army Medical Center, San Antonio, Texas; Naval Medical Center San Diego, San Diego, California; Womack Army Medical Center, Fort Liberty, North Carolina; NHC Annapolis, Annapolis, Maryland; USUHS, Bethesda, Maryland; Uniformed Services University of the Health Sciences, Bethesda, Maryland; Womack Army Medical Center, Fort Liberty, North Carolina; Immunization Healthcare Division, Bethesda, Maryland; Immunization Healthcare Division, Bethesda, Maryland; Immunization Healthcare Division, Bethesda, Maryland; Wake Forest University School of Medicine, Winston-Salem, North Carolina; Naval Medical Center, San Diego, California; Infectious Disease Clinical Research Program, USUHS, Bethesda, Maryland; IDCRP, Bethesda, Maryland; Infectious Disease Clinical Research Program, Department of Preventive Medicine and Biostatistics, Uniformed Services University of the Health Sciences, Bethesda, MD, USA, Bethesda, Maryland; IDCRP, Bethesda, Maryland; Infectious Disease Clinical Research Program, USUHS; Henry M. Jackson Foundation for the Advancement of Military Medicine, Inc., Bethesda, Maryland; Infectious Disease Clinical Research Program, Department of Preventive Medicine and Biostatistics, Uniformed Services University of the Health Sciences, Bethesda, MD, USA, Bethesda, Maryland; Infectious Disease Clinical Research Program, Department of Preventive Medicine and Biostatistics, Uniformed Services University of the Health Sciences, Bethesda, MD, USA, Bethesda, Maryland

## Abstract

**Background:**

Influenza-like illness (ILI) causes significant morbidity. This study aims to compare ILI incidence and severity of current tobacco smokers, former smokers, and non-smokers enrolled in a randomized clinical trial, comparing influenza vaccine effectiveness in a military population over 4 influenza seasons (2018-2022).

**Table 1.**
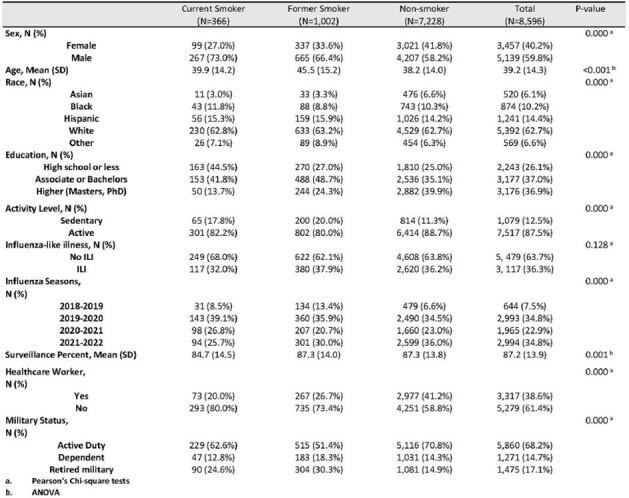
Selected characteristics of the PAIVED study population who responded to >50% of surveillance, by smoking status as reported at enrollment into the study.

**Methods:**

Participants in the Pragmatic Assessment of Influenza Vaccine Effectiveness in the DoD (PAIVED) study responded to weekly surveillance and reported ILIs. Demographic information was collected at enrollment and the Influenza Patient-Reported Outcome (FLU-PRO) instrument was used to measure ILI severity. Analyses included Pearson’s Chi-squared test, ANOVA, and Kruskal-Wallis to compare selected characteristics (Table 1) and FLU-PRO scores based on smoking status. Poisson regression was used to assess the impact of smoking on ILI.

**Table 2:**
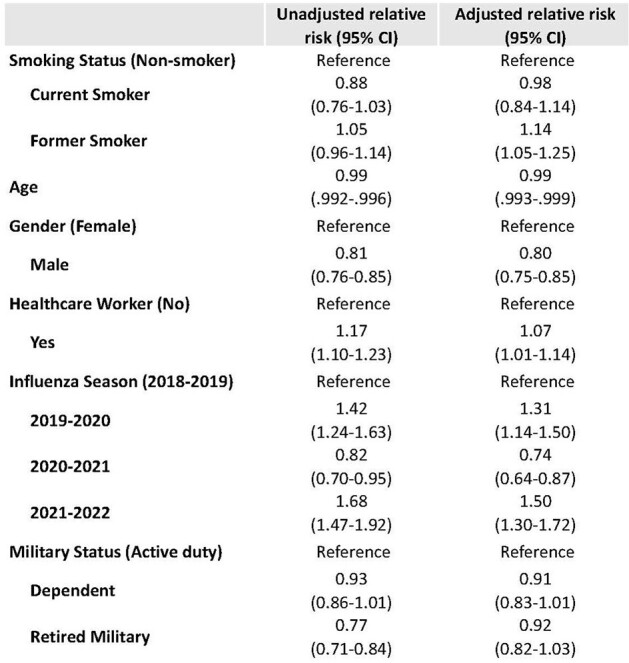
Risk of ILI was compared using a modified Poisson approach. Models were run separately for each variable, and the adjusted model includes all the variables listed in the table.

**Results:**

Over half (56%) of PAIVED participants (8,596/15,432) responded to ≥50% of the ILI surveys and had complete demographic data. The demographic characteristics were different among current smokers, former smokers, and non-smokers (p< 0.001) (Table 1), with smokers more likely to be male and have lower educational levels, among other differences. Over one third (36%) of participants experienced an ILI during the influenza season in which they were enrolled. After adjusting for sex, age, military status, influenza season, and healthcare worker status, former smokers were 14% more likely to report an ILI (aRR 1.14, 95% CI 1.05-1.25) compared to non-smokers, while no difference was observed for current smokers (aRR 0.98, 95% CI 0.84-1.14) (Table 2). Among those with ILIs (N=3,117) who filled out a FLU-PRO survey (N=2,111), FLU-PRO severity scores in the respiratory and eye symptoms domains were different by smoking status, with current smokers reporting the highest scores (p< 0.02) (Table 3).

**Table 3:**
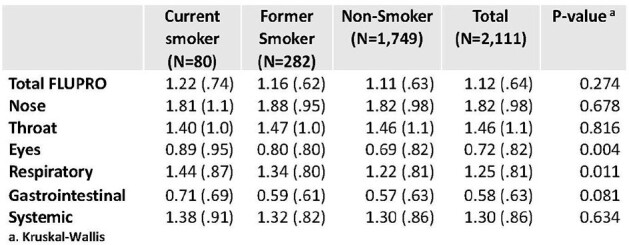
Maximum FLU-PRO scores (mean (SD)) by smoking status for ILIs reported in the PAIVED study.

**Conclusion:**

Former smokers were more likely to report ILI compared to non-smokers. Current and former smokers diagnosed with ILI reported higher respiratory and eye symptom scores than non-smokers. Future analysis will include smoking history (number in the past 7 days, years of smoking), reported electronic cigarette use, and an exploration of asthma and COPD as contributing factors.

**Disclosures:**

**Ryan C. Maves, MD**, AiCuris: Grant/Research Support|Biotest: Grant/Research Support|GeoVax: Grant/Research Support|Shionogi: Advisor/Consultant|Shionogi: Honoraria|Sound Pharmaceuticals: Grant/Research Support **Mark Simons, PhD**, Astrazeneca: Grant/Research Support **Simon Pollett, MBBS**, AstraZeneca: The IDCRP and HJF were funded to conduct an unrelated phase III COVID-19 monoclonal antibody immunoprophylaxis trial as part of US Govt COVID Response **Timothy Burgess, MD, MPH**, AstraZeneca: The IDCRP and HJF were funded to conduct an unrelated phase III COVID-19 monoclonal antibody immunoprophylaxis trial as part of US Govt COVID Response

